# The Effect of Oxygen Supply on the Dual Growth Kinetics of *Acidithiobacillus thiooxidans* under Acidic Conditions for Biogas Desulfurization

**DOI:** 10.3390/ijerph120201368

**Published:** 2015-01-27

**Authors:** Hyeong-Kyu Namgung, JiHyeon Song

**Affiliations:** Department of Civil and Environmental Engineering, Sejong University, 98 Gunja-Dong, Seoul 143-747, Korea; E-Mail: nghgna@naver.com

**Keywords:** biogas desulfurization, sulfur oxidizing bacteria, *Acidithiobacillus thiooxidans*, acidic condition, dual growth kinetic, model simulation

## Abstract

In this study, to simulate a biogas desulfurization process, a modified Monod-Gompertz kinetic model incorporating a dissolved oxygen (DO) effect was proposed for a sulfur-oxidizing bacterial (SOB) strain, *Acidithiobacillus thiooxidans*, under extremely acidic conditions of pH 2. The kinetic model was calibrated and validated using experimental data obtained from a bubble-column bioreactor. The SOB strain was effective for H_2_S degradation, but the H_2_S removal efficiency dropped rapidly at DO concentrations less than 2.0 mg/L. A low H_2_S loading was effectively treated with oxygen supplied in a range of 2%–6%, but a H_2_S guideline of 10 ppm could not be met, even with an oxygen supply greater than 6%, when the H_2_S loading was high at a short gas retention time of 1 min and a H_2_S inlet concentration of 5000 ppm. The oxygen supply should be increased in the aerobic desulfurization to meet the H_2_S guideline; however, the excess oxygen above the optimum was not effective because of the decline in oxygen efficiency. The model estimation indicated that the maximum H_2_S removal rate was approximately 400 ppm/%-O_2_ at the influent oxygen concentration of 4.9% under the given condition. The kinetic model with a low DO threshold for the interacting substrates was a useful tool to simulate the effect of the oxygen supply on the H_2_S removal and to determine the optimal oxygen concentration.

## 1. Introduction

Recently, research and industrial interests in renewable energy sources have increased due to the depletion of natural resources. Among renewable energy sources, biogas is the most reliable and feasible alternative [1,2]. Biogas is commonly generated by anaerobic digestion using organic wastes such as wastewater sludge, food waste, livestock manure, and agricultural by-products. The main components of biogas are methane (CH_4_) and carbon dioxide (CO_2_), and other trace compounds [3]. In order to utilize biogas as an energy source, it needs to be pretreated before use to increase its methane fraction and to remove impurities. 

Biogas commonly contains trace gases such as hydrogen sulfide (H_2_S), ammonia, siloxanes, and so on. The trace components can frequently cause malfunctions and failures in biogas utilization facilities [4]; therefore, the trace components must be eliminated for the effective use of biogas [5]. In particular, the concentration of H_2_S in biogas generally ranges from a few ppm to more than 5000 ppm, and high concentrations of H_2_S may shorten the lifetime of biogas facilities due to corrosion and by-product formation [6]. The criteria regarding the H_2_S concentration for biogas facilities are different in every country. According to a report by Persson *et al.* [7], the guidelines for H_2_S concentration in biogas as a fuel alternative are 23 ppm in Sweden, 5 ppm in Switzerland, 30 ppm in Germany, and 100 ppm in France. In South Korea, the regulatory standard for the H_2_S concentration in biogas should be less than 10 ppm for a use as vehicle fuel.

Common treatment technologies to remove the high concentrations of H_2_S in the gas phase include physical (adsorption, absorption, and dilution), chemical (chemical absorption, neutralization, and combustion) and biological (activated sludge and biofilter) methods [8,9]. Both physical and chemical methods have some drawbacks, such as high operating costs and production of secondary pollutants, particularly when the H_2_S concentration is high [10]. In contrast, the biological methods are cost-effective compared to the physical and chemical processes for the removal of H_2_S from sewage treatment plants [11]. In addition, packed bed biofilters have been widely applied to treat various gas streams because of their relatively low operating costs and minimal production of undesirable by-products [8,12,13]. 

The biological removal of H_2_S mainly relies on sulfur oxidizing bacteria (SOB), which requires oxygen for desulfurization. Elimination capacities (ECs) of H_2_S using aerobic SOB reported in the literature are generally in the range of 36–256 g/m^3^/h depending on the conditions of microbial species and bioreactor operations [14,15,16]. On the other hand, the microbial removal of H_2_S in an anoxic condition using nitrate as an electron acceptor can be another alternative with a maximum EC of 142 g/m^3^/h [17]; however, the anoxic desulfurization requires an addition of nitrate at a high concentration, and the removal capacity declines when the nitrate is limited [18]. As a result, biological methods for the removal of H_2_S have commonly utilized aerobic SOB strains that grew under oxygen-rich conditions [13]. Furthermore, Alcantara *et al.* [19] and Potivichayanon *et al.* [20] also showed that the H_2_S removal rate of aerobic SOB strongly depended on the dissolved oxygen (DO) concentration in the liquid phase, and DO above a threshold value was necessary for the effective biological oxidation of H_2_S. Nevertheless, the oxygen concentration in biogas as a fuel alternative must be lower than 6% (v/v) because of practical and safety problems [4], and this restriction can be one of the major obstacles to biological desulfurization.

In addition to the restriction of oxygen supply, aerobic SOB strains typically showed the highest activity and H_2_S removal rates in neutral pH conditions [21,22]. However, the continuous oxidation of H_2_S results in an accumulation of hydrogen and sulfate ions in the liquid phase, leading to a decline in pH. Low pH may reduce the activity of SOB strains and eventually affect the efficiency of H_2_S removal; therefore, the pH of the liquid phase needs to be adjusted to neutral conditions by adding a basic solution [15]. Comparatively, Takano *et al.* [23] isolated and identified a bacterial strain from volcanic soil, which was capable of removing the sulfur compounds at a low pH. In addition, a SOB stain, *Acidithiobacillus thiooxidans*, showed high activity for the H_2_S oxidation at a pH of 1.5 [22]. Ramirez *et al.* [13] also studied the H_2_S removal rate at pH 2.5 using the same SOB strain, and found the maximum EC of 58 g/m^3^/h. However, H_2_S oxidation kinetics have not studied extensively for the SOB strain at low pH conditions, and oxygen effects on the kinetic parameters and bioreaction rates have yet been determined.

To minimize the oxygen and pH limitations of the biological H_2_S removal, the acidophilic SOB strain, *A. thiooxidans* was adapted for the effective oxidation of H_2_S at an extremely low pH of 2. In this study, the biological parameters of the strain were determined by a series of short-term experiments using a bubble-column bioreactor, and a kinetic model was proposed to adequately represent the experimental results. The kinetic model was evaluated to simulate H_2_S and oxygen utilization rates when both H_2_S and oxygen gases were introduced to the bioreactor. In addition, by applying the proposed kinetics model, an appropriate concentration of free oxygen was suggested for the effective removal of high concentrations of H_2_S under the given conditions.

## 2. Experimental Section 

### 2.1. Microorganisms 

The microbial strain of SOB, *A. thiooxidans*, was originally isolated in a domestic wastewater sludge plant in Korea. The same strain has been extensively utilized in biofilters, and it showed high biodegradation capacity for H_2_S [13,22]. The pure strain of *A. thiooxidans* was maintained in a 250 mL serum bottle containing 50 mL of a sterilized liquid medium. The SOB strain was then cultivated in a 2-L sterile medium in a 5-L flask in the presence of oxygen supplied by continuous aeration. The liquid medium used to cultivate the strain contained sodium thiosulfate (Na_2_S_2_O_3_) 5.1 g/L, K_2_HPO_4_ 2.0 g/L, KH_2_PO_4_ 2.0 g/L, NH_4_Cl 0.4 g/L, MgCl_2_·7H_2_O 0.2 g/L, FeSO_4_·7H_2_O 0.01 g/L, and yeast extract 0.01 g/L. The pH of the medium was adjusted to 2 using HCl. Prior to the kinetic parameter estimation and bioreactor experiment, sodium thiosulfate was not added to the liquid medium, and a gaseous stream containing oxygen and H_2_S at a concentration of 100 ppm was supplied to the culture as a bubble stream through a diffuser for two weeks to minimize the bacterial adaptation period. When the microbial density of the SOB strain reached approximately 1000 mg-dry weight/L, the culture was diluted with the thiosulfate-free nutrient medium and transferred to a bubble-column bioreactor described below. 

### 2.2. Bubble-Column Bioreactor

In this study, a lab-scale bioreactor consisted of a column with a cross-sectional area of 0.0025 m^2^ and an effective height of 0.2 m, corresponding to a working volume of 0.5 L. As a surrogate of biogas, the gas stream used in this study was mainly composed of nitrogen instead of methane due to safety issues. A preliminary test showed that the microbial activity of the strain in the presence of methane was not substantially different from that of nitrogen (data not shown). The gas stream containing 1% H_2_S was combined with pure nitrogen and oxygen, and the fraction of each gas component in the mixture controlled by a gas flow meter depending on the target H_2_S and oxygen concentrations. The mixed gas stream was supplied to the bottom of the bioreactor through a fine-bubble sparger. Gas sampling ports were installed in the inlet and the outlet lines to measure the concentrations of H_2_S in the gas phase, and liquid samples were also taken from a port located at the middle of the column. 

### 2.3. Bioreactor Experiments

First, bioreactor experiments were performed to determine the kinetic parameters of the SOB strain. The pre-cultivated SOB strain was initially adjusted to a microbial density of 220 ± 8 mg-dry weight/L. The experiments were performed by measuring the removal rates of H_2_S and the growth rates of the microbial strain under various oxygen and H_2_S concentrations supplied to the bioreactor. The gas-phase concentrations of H_2_S tested in this kinetic study were 500, 1000, 1500, 2000, and 3000 ppm at the overall gas flowrate of 0.5 L/min, corresponding to a gas retention time (GRT) of 1 min. The DO concentrations in the liquid medium were varied at 0.5, 1, 1.5, 2, 3, and 6 mg/L by adjusting the volumetric flowrate of oxygen in the influent gas stream. The bioreactor was operated for 48 h at the same gas flow rate in each experiment. The microbial density and the H_2_S removal rate were measured every 12 h. 

Following the kinetic study, a series of short-term bioreactor experiments was conducted to validate the kinetic model and to determine the activity of the SOB strain using the same bubble-column bioreactor. For each short-term test, the initial microbial density was 500 ± 10 mg-dry weight/L by adjusting the pre-acclimated SOB culture with the fresh medium. The GRTs were changed at 1, 5 and 10 min, while the H_2_S concentration in the mixed gas stream was fixed at 1000 ppm. The bioreactor performance was generally stabilized within two hours, and the H_2_S removal rates were quantified using four gas samples collected at a two-hour interval after the pseudo-steady state reached. 

### 2.4. Analytical Methods 

To determine the removal efficiency of H_2_S, the influent and effluent H_2_S concentrations in the gas phase were directly measured using an electrochemical detector (GFM series, GASDATA, Conventry, UK).The gas detector was calibrated using three-point H_2_S gas standards. The liquid samples withdrawn from the sampling port of the bioreactor were analyzed for the DO concentration (YSI-5000, YSI, Yellow Springs, OH, USA), and pH (PB-11, Sartorius, Goettingen, Germany). The microbial density in the liquid medium was gravimetrically quantified by dry weight. The sulfate ion concentration in the liquid was analyzed using an ion chromatography apparatus (792 IC, Metrohm, Herisau, Switzerland) equipped with an A-supp-5 column. The calibration curve of the sulfate ion for the chromatographic measurement was made using five standard reagents in a range of 0–100 mg-S/L. The liquid-phase concentration of H_2_S was quantified using a specialized analyzer (H_2_S Analysator, ECH Elektrochemie Halle GmbH, Halle, Germany), in which the liquid sample was first treated with 1% phosphoric acid solution, and then all the dissolved H_2_S was instantaneously evaporated at 150 °C in a heating block. The H_2_S concentration in the evaporated gas was measured by a gas sensor unit in the analyzer, and the liquid-phase concentration was calculated using the overall mass of H_2_S in the liquid sample. The analyzer was calibrated using known H_2_S standards in a range of 0–10 mg-S/L, and the detection limit was found to be 0.01 mg-S/L. 

## 3. Model Development

### 3.1. Mass Balance Equations for the Liquid Phase

The changes in concentration of the target compounds in the liquid phase were estimated using coupled mass balances with simplified assumptions. The bioreactor applied in this study was assumed to be homogeneous and completely mixed. Since there was no liquid medium addition or withdrawal during the kinetic experiments, the target compounds, *i.e.*, H_2_S and DO, were mass-transferred only by absorption from the gas phase into the liquid, and they were finally utilized by the microbial oxidation. A basic mass balance model can be expressed as Equation (1) below:

(1) $$V_{L}\frac{{\text{dC}}_{L}}{\text{dt}}=R_{\text{ab}}+R_{\text{bio}}$$

where, V_L_ is the effective liquid volume of the bioreactor (L), and C_L_ is the concentration of each compound in the fluid phase (mg/L). R_ab_ and R_bio_ represent the rates of removal (mg/min) by absorption and biodegradation, respectively. The absorption term in Equation (1) is expressed by a mass transfer equation between the gas and liquid phases:

(2) $$R_{\text{ab}}=\;K_{L}a\cdot (\frac{C_{\text{Gin}}}{H}-C_{L})$$

where, C_Gin_ is the concentration of the compound in the influent gas stream (mg/L), K_L_a is the mass transfer coefficient by the bubble swarm (min^−1^), and H is the Henry’s law constant (‒).

In a previous study using the same bubble diffuser and bioreactor schematics, the mass transfer coefficient for oxygen (K_L_a_O_) was experimentally determined to be 1.42 min^−1^ [24]. The values of K_L_a for H_2_S, K_L_a_S_, were then estimated based on the penetration model, Equation (3), using the liquid phase diffusivities of oxygen and H_2_S. The K_L_a_S_ for the bubble bioreactor was calculated as 1.16 min^−^^1^:

(3) $$\frac{K_{L}a_{S}}{K_{L}a_{O}}={\left(\frac{D_{S}}{D_{O}}\right)}^{0.5}$$

where D_S_ and D_O_ are the diffusion coefficients of H_2_S and DO in the liquid (cm^2^/s), respectively. 

The biodegradation rate by the SOB strain is presented in the following equation:

(4) $$R_{\text{bio}}=\mu_{\text{max}}\cdot V_{L}\cdot \frac{X}{Y_{X}}\cdot \gamma_{\text{bio}}$$

where, μ_max_ is the maximum specific growth rate (min^−^^1^), Y_X_ is the yield coefficient of microorganisms using target substrates (mg-dry weight/mg-substrate), X is the microbial density in the liquid phase (mg-dry weight/L), and γ_bio_ represents the biomass growth kinetic (‒). 

A microbial growth kinetic model is generally constructed using the single Monod equation in which a single substrate is utilized. Furthermore, a modified growth kinetic model using the dual Monod equation is commonly used when dual substrates are introduced [25,26]. However, the dual Monod equation is known to often fail to precisely predict microbial growth when multiple substrates are interacting with others. Omar *et al.* [27] applied Monod, Logistic, and Gompertz equations to more accurately predict microorganism growth rates for interacting substrates. In this study, a modified Monod-Gompertz kinetic, which combined the Monod equation for H_2_S and the Gompertz equation for DO, was proposed to simulate the microbial growth rate of the SOB strain. A preliminary experiment showed that the H_2_S removal of the strain strongly depended on the DO concentration being above a threshold (data not shown). The modified Monod-Gompertz is expressed as follows:

(5) $$\gamma_{\text{bio}}=\frac{C_{\text{LS}}}{K_{S}+C_{\text{LS}}}\cdot \text{exp}\left[-\text{exp}\left(\frac{K_{O}-C_{\mathrm{LO}}}{K_{O}/2}\right)\right]$$

where, K_S_ is the half saturation constant of H_2_S (mg/L), C_LS_ is the H_2_S concentration in the liquid (mg/L), K_O_ is the kinetic constant of oxygen for the microbial growth in the liquid (mg/L), and C_LO_ is the DO concentration in the liquid (mg/L). The overall biodegradation rate of the SOB strain in the bioreactor can be expressed as follows by combining Equations (4) and (5):

(6) $$R_{\text{bio}}=\mu_{\text{max}}\cdot V_{L}\cdot \frac{X}{Y_{X}}\cdot \frac{C_{\text{LS}}}{K_{S}+C_{\text{LS}}}\cdot \text{exp}\left[-\text{exp}\left(\frac{K_{O}-C_{\text{LO}}}{K_{O}/2}\right)\right]$$

The yield coefficient of the SOB strain by the H_2_S oxidation, Y_X/S_, can be calculated using the following Equation (7). In addition, the microbial yield of the SOB strain for oxygen, Y_X/O_, can be calculated using the stoichiometric ratio between oxygen and sulfur in the oxidation reaction, similarly to the yield relationship of oxygen and nitrogen (*i.e.*, Y_X/O_ = Y_X/N_/(4.57 − Y_X/N_)) in an activated sludge model [28]. The relationship of oxygen and sulfur indicates that the yield coefficient for oxygen is expressed as Y_X/O_ = Y_X/S_/(2 − Y_X/S_) in a molar ratio basis. In a preliminary test, a conversion factor for the dry cell weight and the stoichiometric molar ratio was determined, and the microbial yield of the SOB for oxygen can be derived as given by Equation (8) below:

(7) $$Y_{X/S}=\frac{\text{dX}}{{\text{dC}}_{\text{LS}}}$$

(8) $$Y_{X/O}=\frac{Y_{X/s}}{0.22-Y_{X/s}}$$

By combining all the equations mentioned above, the overall mass balances for H_2_S and for DO in the liquid phase of the bioreactor can be expressed with Equations (9) and (10), respectively:

(9) $$\frac{{dC}_{LS}}{dt}=K_{L}a_{S}(\frac{C_{\text{GSin}}}{H_{S}}-C_{\text{LS}})-\frac{1}{Y_{X/S}}\cdot \mu_{\text{max}}\cdot X\cdot \frac{C_{\text{LS}}}{K_{S}+C_{\mathrm{LS}}}\cdot \text{exp}\left[-\text{exp}\left(\frac{K_{O}-C_{\text{LO}}}{K_{O}/2}\right)\right]$$

(10) $$\frac{{\text{dC}}_{\text{LO}}}{\text{dt}}=K_{L}a_{O}\cdot (\frac{C_{\text{GOin}}}{H_{O}}-C_{\text{LO}})-\left(\frac{\mathrm{0.22}-Y_{X/S}}{Y_{X/S}}\right)\cdot \mu_{\text{max}}\cdot X\cdot \frac{C_{\text{LS}}}{K_{S}+C_{\mathrm{LS}}}\cdot \text{exp}\left[-\text{exp}\left(\frac{K_{O}-C_{\text{LO}}}{K_{O}/2}\right)\right]$$

where, C_GSin_ and C_GOin_ are the influent concentrations of H_2_S and oxygen in the gas stream introduced to the bioreactor, respectively (mg/L).

### 3.2. Mass Balance Equations for the Gas Phase

The concentration changes of target compounds, *i.e.*, H_2_S and oxygen, in the gas phase were also estimated using coupled mass balances with the same assumptions described above. Since the bubble swarm rose rapidly and was mixed well, the gaseous concentrations were assumed to have neither heterogeneous distributions nor spatial gradients. The target compounds were mass-transferred both by advection in the gas stream and by absorption from the gas phase into the liquid. The overall mass balance equations for H_2_S and oxygen in the gas phase are derived as Equations (11) and (12), respectively:

(11) $$\frac{{\text{dC}}_{\text{GSout}}}{\text{dt}}=\frac{Q_{G}}{V_{G}}\cdot \left(C_{\text{GSin}}-C_{\text{GSout}}\right)-K_{L}a_{S}\cdot (\frac{C_{\text{GSin}}}{H_{S}}-C_{\text{LS}})$$

(12) $$\frac{{\text{dC}}_{\text{GOout}}}{\text{dt}}=\frac{Q_{G}}{V_{G}}\cdot {(C}_{\text{GOin}}-C_{\text{GOout}})-K_{L}a_{O}\cdot (\frac{C_{\text{GOin}}}{H_{O}}-C_{\text{LO}})$$

where, V_G_ is the volume of the gas bubbles in the bioreactor (L), and C_GSout_ and C_GOout_ are the effluent concentrations of H_2_S and oxygen in the gas stream, respectively (mg/L).

### 3.3. Model Parameter Estimation and Prediction

To estimate the kinetic parameters, nonlinear curve fitting was performed using the 4th-order Runge-Kutta iteration sequence. The kinetic parameters for the dual substrate experiments were determined using the best fit method where the sum of squared errors between actual experimental data and simulated values for a set of estimated parameters were minimized. In this modeling study, the coupled differential equations, *i.e.*, Equations (9), (10), (11) and (12), were solved simultaneously using the numerical method. The main model variables included microbial density, gas retention time (GRT), and influent concentrations of oxygen and H_2_S. The H_2_S removal rate and the required oxygen concentration were predicted using the determined model variables and parameters.

## 4. Results and Discussion

### 4.1. Growth Kinetic Model and Parameter Estimation

The growth kinetic experiments using the pure SOB culture, *A. thiooxidans*, were carried out under the extremely acidic conditions of pH 2 in this study. In low pH conditions, all the sulfide species exist in the non-ionized acidic form, *i.e.*, H_2_S. In addition, a preliminary study on a mass balance analysis for the H_2_S oxidation showed that greater than 95% of the introduced H_2_S was converted to sulfate ion, SO_4_^2−^, even in a limited oxygen condition (data not shown), indicating that the production of intermediates such as elemental sulfur or thiosulfate was minimal by the SOB strain. Contrarily, several studies for biogas desulfurization have reported the accumulation of elemental sulfur as the end-product when oxygen was limited for the H_2_S oxidation [17,19,21]. Since the sulfate ion was the predominant end-product in this study, the production of the intermediates was not taken into account for the H_2_S oxidation. The growth rate of the SOB strain and the H_2_S removal rate were measured as a function of the concentrations of H_2_S and oxygen introduced into the bioreactor. 

First, the kinetic experiments were performed to measure the specific growth rate of the SOB by varying the concentration of H_2_S in the gas stream at 500, 1000, 1500, 2000, and 3000 ppm, while the DO concentration in the liquid phase was maintained at 5.9 ± 0.3 mg/L to minimize the oxygen effect. The corresponding concentrations of H_2_S in the liquid phase were measured as 0.16, 0.63, 1.79, 2.79, and 4.62 mg-S/L, respectively, in the presence of the active microbial culture. The liquid-phase abiotic concentrations of H_2_S calculated using the Henry’s law constant were 1.64, 3.27, 4.91, 6.54, and 9.82 mg-S/L, respectively, corresponding to the gaseous concentrations. The differences between the biotic and abiotic concentrations of H_2_S indicated that the SOB strain was sufficiently active to oxidize H_2_S in the acidic medium.

**Figure 1:**
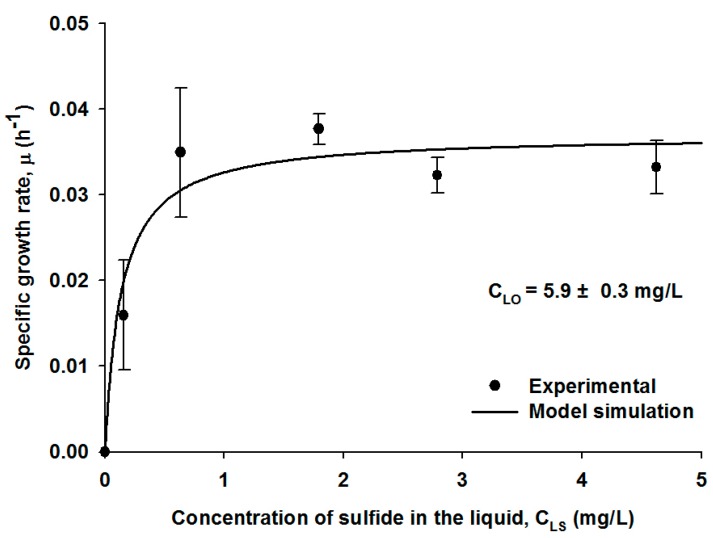
The specific growth rates of the SOB strain at different H_2_S concentrations in the liquid medium. The symbols show averaged experimental data with standard deviations, and the line illustrates model fitting.

The specific growth rate of the SOB strain obtained in the H_2_S loading experiments showed a typical Monod-type pattern, as illustrated in Figure 1. A best-fit procedure was performed using the experimental data and Equation (6) without the oxygen effect, and the maximum specific growth rate, μm, was estimated as 0.037 h^−^^1^, which was similar to the value of 0.041 h^−^^1^ for *Thiobacillus* sp. reported in the literature [29]. Furthermore, the half saturation constant, K_S_, was estimated to be 0.15 mg-S/L, which was very lower than the reported values of 8.9–11 mg/L for similar SOB species [19,26]. The low half saturation constant in this study indicates that the microbial strain grew well even at a low concentration of H_2_S. These results also imply that the microbial activity of the SOB strain used in this study was maintained and even enhanced for the H_2_S biodegradation in the extremely low pH. 

The oxygen concentration in the liquid medium is an essential element for the aerobic biodegradation, and it must be considered with the main substrate, *i.e.*, H_2_S, for the microbial growth kinetic relationship. Similarly, nitrification, the oxidation of ammonium to nitrate using DO by aerobic nitrifying bacteria, is an example for a dual substrate kinetic, and the nitrification and subsequent microbial growth did not take place at a threshold DO concentration of 0.5 mg/L [25]. In order to determine both a threshold DO concentration and a DO effect on the kinetics for the H_2_S oxidation by the SOB strain, growth rate experiments were also performed by varying the flowrate of oxygen introduced into the gas phase, while the experimental conditions including the pH and microbial density were kept the same as in the previous H_2_S loading experiments, and the H_2_S concentration introduced to the gas stream was maintained at approximately 500 ppm corresponding to the liquid-phase concentration of 0.18 ± 0.02 mg/L. 

**Figure 2:**
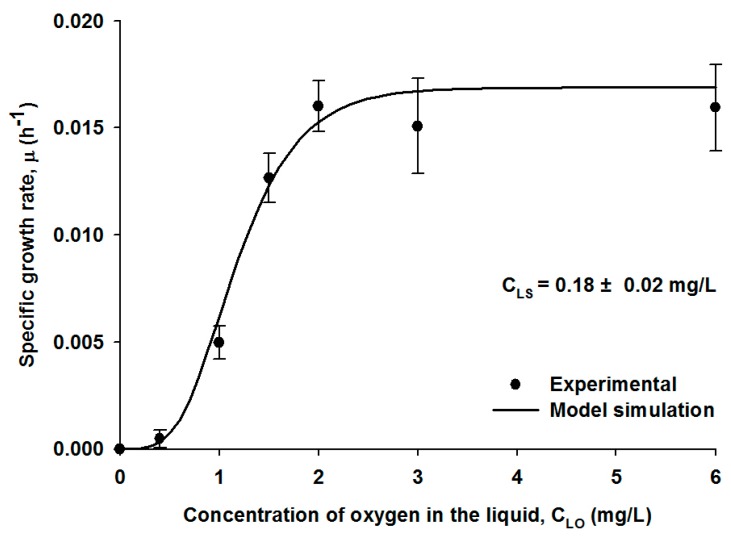
The specific growth rates of the SOB strain as a function of the oxygen concentrations in the liquid medium. The symbols show averaged experimental data with standard deviations, and the line illustrates model fitting.

The specific growth rate of the SOB strain as a function of the DO concentration showed a sigmoidal shape as illustrated in Figure 2. Growth and biodegradation of the SOB strain was not observed at a DO concentration of below 0.4 mg/L, the threshold concentration. The growth rate exponentially increased with increasing DO concentration in a range of 0.5–2.0 mg/L. At DO concentrations greater than 2.0 mg/L, the microbial growth rate remained unchanged with the maximum rate of 0.017 ± 0.003 h^−1^. Consequently, the DO of 2.0 mg/L was the minimum concentration required to obtain the maximal activity and H_2_S removal. 

The microbial growth kinetic with the DO effect was estimated using Equation (6). The microbial growth kinetics for binary substrates has typically been described using the dual-Monod equation [25,26]; the dual-Monod kinetics cannot predict either the sigmoidal pattern or the low threshold where the microbial growth rate becomes almost zero. As a result, the modified Monod-Gompertz kinetic, Equation (5), was suggested in this study. The model simulation and the best-fit sequence using the modified Monod-Gompertz kinetic were performed, and the numerical result is illustrated as a solid line in Figure 2. The half saturation constant of DO, K_O_ in the Monod-Gompertz equation represents an inflection point where the slope of the microbial growth rate changes from positive to negative; and it was estimated to be 1.10 mg/L. 

The yield coefficient for the H_2_S utilization, Y_X/S_, is one of the important parameters for the kinetic model. Another bacterial growth experiment was performed at a DO concentration of 6.0 mg/L and an initial microbial density of 200 ± 20 mg-dry weight/L. The yield coefficient was calculated to be 0.093 mg-dry weight/mg-S using Equation (7), which was similar to another yield value of 0.09 mg/mg for an SOB strain reported in the literature [26]. In cases of multiple, interacting substrates, the other yield coefficient for the secondary substrate can be estimated using that of the primary substrate [25], and Equation (8) was derived and used to estimate the yield coefficient for oxygen, Y_X/O_, at 0.732 mg/mg. All the kinetic model parameters estimated in this study are listed in Table 1. 

**Table 1:** Kinetic model parameters used in this study.

Parameters		Values	Bioreactors	Reference
Estimated	μ_max_	0.037	1/h	this study
	K_S_	0.15	mg-S/L	this study
	K_O_	1.10	mg-O/L	this study
	Y_X/S_	0.093	mg-dry weight/mg-S	this study
	Y_X/O_	0.732	mg-dry weight/mg-O	this study
Known	H_S_	0.427	-	[30]
	H_O_	32.3	-	[30]
	D_S_	0.0000161	cm^2^/s	[30]
	D_O_	0.0000240	cm^2^/s	[30]
	K_L_a_O_	1.42	1/min	[24]

### 4.2. Model Validation and Simulation

The microbial removal of H_2_S in biogas requires DO at a concentration greater than the threshold, but the oxygen supply in the gas phase has an upper limit, *i.e.*, 6% by volume, due to economic and safety issues [5]. Consequently, the appropriate oxygen concentration required for biogas desulfurization must be carefully selected to ensure the effective removal of H_2_S under diverse operating conditions. In this study, the kinetic model was validated using the data from the short-term bioreactor experiments. Furthermore, the model simulation was used to determine the optimal oxygen concentration required at different gas retention times and influent H_2_S concentrations.

#### 4.2.1. Effects of Gas Retention Times (GRTs)

**Figure 3:**
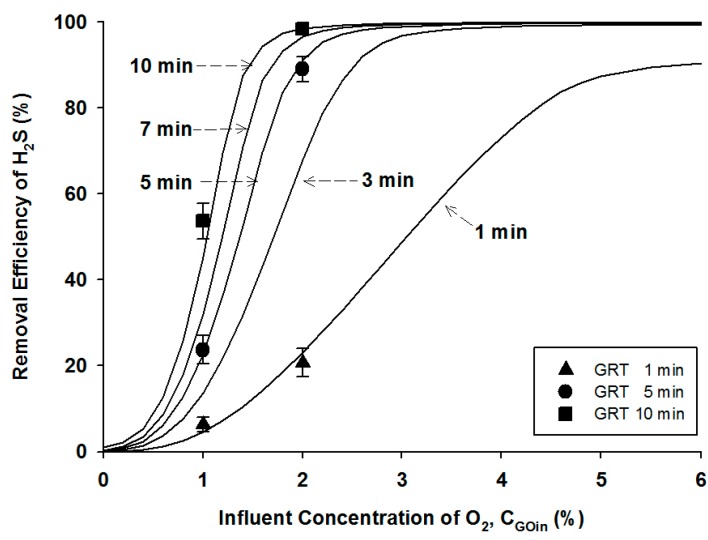
Experimental data (symbols) and model simulation results (lines) at different gas retention times as a function of the influent oxygen content.

The experimental results obtained from the short-term bioreactor operation were used to evaluate the numerical model using the coupled mass balance Equations (10), (11), (12), and (13) and its parameters described above. The experimental and model simulation results for the H_2_S oxidation as a function of the oxygen content in the influent gas stream was illustrated in the Figure 3. In the short-term bioreactor experiments, the H_2_S concentration in the influent gas stream was remained constant at 1000 ± 45 ppm, and the initial microbial density was 500 ± 20 mg/L, while three different GRTs of 1, 5, and 10 min were applied at the influent oxygen contents of 1% and 2%, respectively. At the oxygen content of 1% and the GRT of 1 min, the H_2_S removal efficiency was less than 5%, indicating that the biological oxidation of H_2_S was minimal. When the oxygen content in the gas stream increased to 2% while the other conditions remained unchanged, the H_2_S removal efficiency increased slightly to approximately 20%. As expected, the longer GRT, the higher removal efficiency at the given oxygen contents. At the longest GRT of 10 min, the H_2_S removal efficiencies were found to be 54% and 98% at the influent oxygen contents of 1% and 2%, respectively. These results imply that the oxygen supply be one of the most critical parameters for aerobic bio-desulfurization as the H_2_S loading increases. 

To determine the GRT required to meet the H_2_S concentration in the effluent gas stream and the oxygen supply, the numerical simulation using the kinetic model was performed. In the model simulation, as the same as the experimental conditions, an influent concentration of H_2_S of 1000 ppm and the initial microbial density of 500 mg/L were applied, and the H_2_S removal efficiencies were calculated at the GRTs of 1, 3, 5, 7 and 10 min as a function of the oxygen content in the influent gas stream, as illustrated in Figure 3. The model simulations agree well with the experimental results at the given conditions, indicating that the numerical model applied in this study was suitable for the prediction of the performance of the bioreactor using the SOB strain at the low pH condition.

Similarly to the experimental results, the model prediction shows that the H_2_S removal efficiency increased with increasing the GRT at a given oxygen content. To meet the regulatory standard for biogas utilization, e.g., the H_2_S concentration of 10 ppm in Korea, the minimum requirements of the oxygen supply in the influent gas stream were estimated to be 4.3, 3.1, 2.7 and 2.3% at the GRT of 3, 5, 7, and 10 min, respectively. Note that the regulatory H_2_S standard could not be met in the bioreactor operating at the GRT of 1 min, even at the oxygen concentration of 6% or higher. This indicates that the influent oxygen concentration should be controlled in a range of 2%–6% for the effective removal of H_2_S, and a GRT of more than 3 min needs to be selected to achieve the design purpose in this bioreactor configuration. 

#### 4.2.2. Effects of Microbial Density

**Figure 4:**
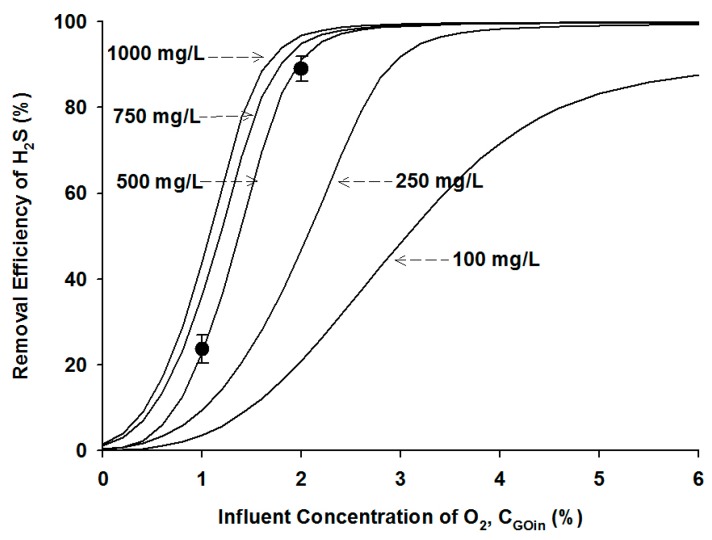
Experimental data (symbols) and model simulation results (lines) at different microbial densities as a function of the influent oxygen content.

The changes in H_2_S removal efficiency at various microbial densities and influent oxygen contents were simulated, and the simulation results and experimental data are illustrated in Figure 4. The experimental data was obtained from a bioreactor operating condition at the influent H_2_S concentration of 1000 ppm and the GRT of 5 min. At the lowest microbial density of 100 mg/L simulated in this study, the H_2_S removal efficiency greater than 99%, *i.e.*, the effluent H_2_S concentration of 10 ppm, could not be met even at the highest oxygen contents of 6%. Therefore, the low microbial density was not feasible and applicable for the biological process to achieve the effective removal of H_2_S in biogas. When the microbial density increased to 250 mg/L in the simulation, the H_2_S removal efficiency increased, and the effluent H_2_S concentration of 10 ppm would be achieved at the oxygen content of greater than 5%. At the microbial densities of higher than 500 mg/L, the changes of the H_2_S removal efficiency were not substantial as a function of the oxygen content as shown in Figure 4, and the effluent H_2_S standard would be met at the oxygen content of 3% or higher. 

#### 4.2.3. Effects of Influent H_2_S Concentration

**Figure 5:**
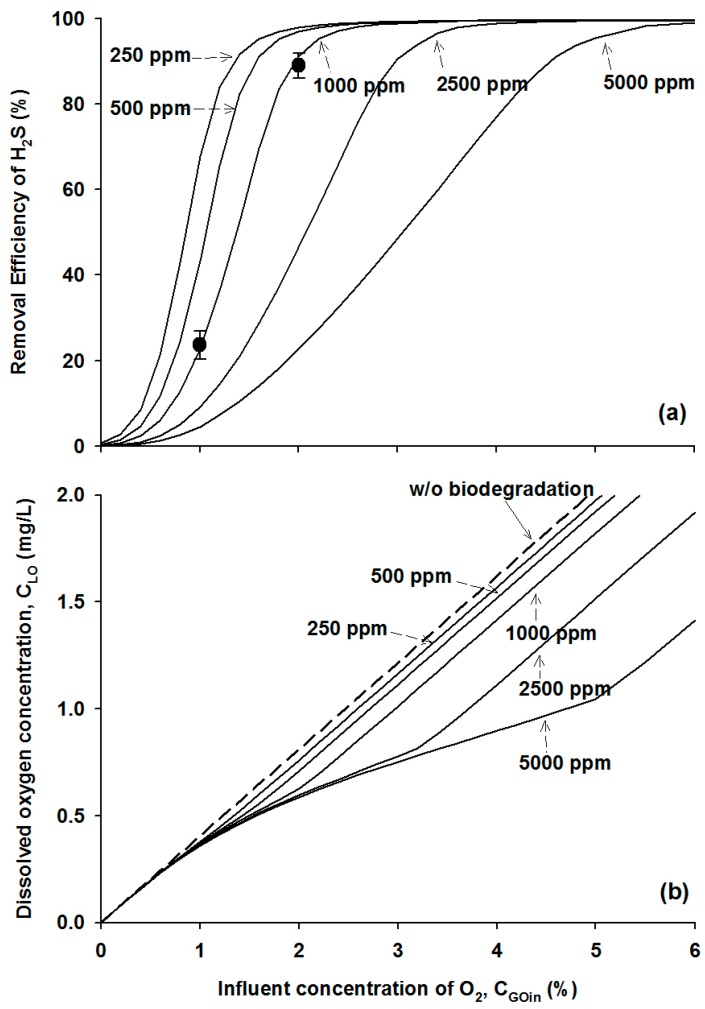
Experimental data (symbols) and model simulation results (lines) of (**a**) the H_2_S removal efficiency and (**b**) dissolved oxygen concentration at different influent oxygen contents and H_2_S concentrations.

The H_2_S concentration in biogas varies widely depending on the source of the anaerobic digestion. In this study, the H_2_S removal efficiencies were numerically determined at the influent H_2_S concentrations of 250, 500, 1000, 2500 and 5000 ppm as a function of the oxygen content introduced into the influent gas stream illustrated in Figure 5a, while the other simulation parameters remained unchanged at the GRT of 5 min and the initial microbial density of 500 mg/L. Again, to meet the H_2_S regulatory standard of 10 ppm in the effluent gas stream, the simulation estimated that the oxygen fractions of 2.6, 2.8, 3.2, and 4.2% (v/v) were required at the influent H_2_S concentrations of 250, 500, 1000, and 2500 ppm, respectively. These results imply that the biological oxidation of H_2_S at a concentration of less than 2500 ppm can occur easily when the gas stream is mixed with oxygen at a content less than 5% in this bioreactor operation. However, at the H_2_S concentration of 5000 ppm, this biological method could not meet the regulatory standard for the effluent stream even when the oxygen content was increased to more than 6%, the practical guideline for biogas utilization. As a result, the operating conditions of the bioreactor in this study were not suitable for the removal of the high concentration of H_2_S of more than 5000 ppm, and the gas retention time and/or the microbial density should be increased to achieve the required removal efficiency.

The DO concentrations in liquid phase at different H_2_S and oxygen loading conditions were compared using the model simulations as illustrated in Figure 5b. In cases of abiotic operation without the active SOB strain, the DO concentration in the liquid increased in a linear manner up to 2.0 mg/L in a range of the influent oxygen fraction from 0% and 5% as delineated as a dotted line in Figure 5b. In the presence of the active SOB strain, the DO concentration decreased due to the biological utilization, and the amount of utilized oxygen increased as both the inlet H_2_S and the biodegradation rate increased. It is interesting to note that, under the condition of an oxygen supply of less than 6% simulated in this study, the DO concentration in the bioreactor medium was always lower than 2.0 mg/L where the biodegradation kinetic rate was sensitive, as shown in Figure 3. 

**Figure 6:**
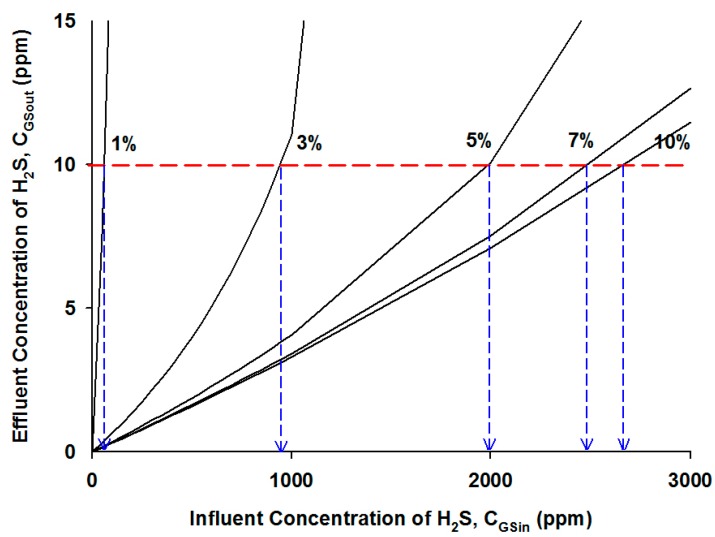
Model simulation results of the H_2_S concentrations at the effluent stream at different influent H_2_S concentrations and oxygen contents.

**Figure 7:**
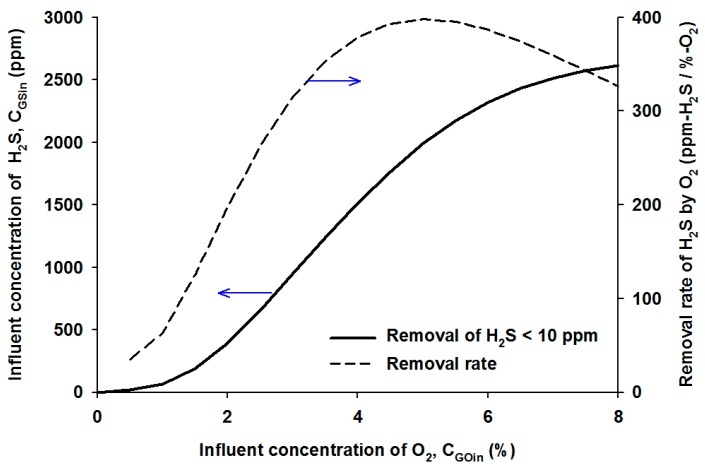
The highest inlet concentration of H_2_S and the specific removal rate of H_2_S as a function of the inlet oxygen content.

Figure 6 is presented to show the simulation results on the effect of the oxygen supply in the gas stream on the effluent H_2_S concentration. The horizontal dotted line represents the regulatory H_2_S concentration of 10 ppm, and the vertical arrows indicate the highest inlet concentration of H_2_S that can be treated at the given oxygen content. For instance, the highest concentration of H_2_S to meet the regulatory standard was approximately 2000 ppm at an oxygen content of 5%. Comparatively, when the influent concentration of H_2_S increases to more than 2500 ppm, the bioreactor required the oxygen supply at more than 10%, which was practically unsafe and infeasible. The same simulation results are also illustrated in Figure 7, which shows the highest inlet concentration of H_2_S that can be treated to meet the standard of 10 ppm as a function of the inlet oxygen content.

In order to determine an appropriate oxygen supply for design and practical purposes, the specific removal of H_2_S normalized by the required oxygen concentration was calculated and given in Figure 7 as the dotted line. At low oxygen supply conditions, the specific removal of H_2_S, *i.e.*, the allowable H_2_S concentration per oxygen, increased with increasing oxygen content.

The specific removal of H_2_S showed the highest value of approximately 400 ppm/%-O_2_ at the influent oxygen content of 4.9%. This value implies that the H_2_S biodegradation should be operated in the condition of an optimal oxygen supply, and the excess oxygen would not be efficiently utilized at an oxygen content of more than the optimal point. These simulation results can change depending on the bioreactor volume, GRT, and microbial density; nevertheless, the introduction of oxygen above the optimal concentration is not practically effective in the bioreactor because of the decline in oxygen efficiency. 

## 5. Conclusions 

In this study, the dual growth kinetic model was proposed for the SOB stain, *A. thiooxidans* capable of degrading H_2_S under the extremely acidic condition of pH 2, and the model parameters for the removal of H_2_S in biogas were determined using the experimental data obtained from the short-term bioreactor operation. The modified Monod-Gompertz kinetic, which combined the Monod equation for the H_2_S oxidation and the Gompertz for the oxygen effect, was developed. The maximum growth rate of the SOB strain at 0.037 h^−1^ and the half saturation constant of H_2_S at 0.15 mg-S/L were estimated, which indicated that the SOB strain was effective for the H_2_S degradation at the acidic condition. The experimental results showed, however, that the H_2_S degradation rate was highly sensitive at the DO concentrations of less than 2.0 mg/L. Using the modified Monod-Gompertz equation proposed in this study, the kinetics with the low DO threshold for the dual and interacting substrates could successfully be predicted.

The effect of the oxygen supply on H_2_S removal efficiency was determined by the short-term bioreactor experiments, and the optimal oxygen concentration was suggested by the model simulation. The influent oxygen concentration should be controlled in a range of 2%–6% for the effective removal of H_2_S at the low to moderate loading conditions. However, the H_2_S regulatory standard could not be met even when the oxygen supply was greater than 6%, the practical guideline, when the H_2_S loading was high, *i.e.*, the short GRT of 1 min and the H_2_S inlet concentration of 5000 ppm, as well as when the microbial density was low less than 250 mg/L. As a result, the oxygen supply as well as the other bioreactor operating conditions including the GRT and the microbial density should be increased to meet the H_2_S standard. The introduction of oxygen above the optimal content, however, might not be feasible, as the utilization efficiency of oxygen declined. The model estimation indicted that the maximum H_2_S removal rate was approximately 400 ppm/%-O_2_ at the influent oxygen concentration of 4.9% in the given conditions of this study. 

## Nomenclature

C_in_: the concentration of the compound introduced to the bioreactor (mg/L)
C_Gin_: the concentration of the compound entering the bioreactor in the gas stream (mg/L)
C_GSin_: the influent concentration of H_2_S entering the bioreactor in the gas stream (mg/L)
C_GOin_: the influent concentration of oxygen in the gas stream (mg/L)
C_GSout_: the effluent concentration of H_2_S exiting the bioreactor in the gas stream (mg/L)
C_GOout_: the effluent concentration of oxygen in the gas stream (mg/L)
C_L_: the concentration of the compound in the liquid phase (mg/L)
C_LS_: the H_2_S concentration in the liquid phase (mg/L)
C_LO_: the oxygen concentration in the liquid Phase (mg/L)
D_S_: the diffusion coefficient of H_2_S in the liquid (cm^2^/sec)
D_O_: the diffusion coefficient of oxygen in the liquid (cm^2^/sec)
H: the Henry’s law constant (-)
H_S_: the Henry’s law constant of H_2_S (-)
H_O_: the Henry’s law constant of oxygen (-)
K_L_a: the mass transfer coefficient (min^-1^)
K_L_a_S_: the mass transfer coefficient of H_2_S (min^-1^)
K_L_a_O_: the mass transfer coefficient of oxygen (min^-1^)
K_S_: the half saturation constant of H_2_S (mg-S/L)
K_O_: the kinetic constant of oxygen (mg-O/L)
μ: the specific growth rate (min^-1^)
μ_max_: the maximum specific growth rate (min^-1^)
Q: the gas flow rate (L/min)
R_ab_: the compound removal rate by absorption (mg/min)
R_bio_: the compound removal rate by biodegradation (mg/min)
γ_bio_: the biomass growth kinetic (-)
V_G_: the volume of the gas in the liquid (L)
V_L_: the effective liquid volume (L)
X: the biomass density in the liquid (mg-dry weight/L)
Y_X/S_: the yield coefficient of the biomass depending on H_2_S (mg-dry weight/mg-S)
Y_X/O_: the yield coefficient of the biomass depending on oxygen (mg-dry weight/mg-O)
